# Concordance and limits between transcutaneous and arterial carbon dioxide pressure in emergency department patients with acute respiratory failure: a single-center prospective observational study

**DOI:** 10.1186/s13049-015-0120-4

**Published:** 2015-05-17

**Authors:** Xavier Bobbia, Pierre-Géraud Claret, Ludovic Palmier, Michaël Robert, Romain Genre Grandpierre, Claire Roger, Patrick Ray, Mustapha Sebbane, Laurent Muller, Jean-Emmanuel de La Coussaye

**Affiliations:** Pôle Anesthésie Réanimation Douleur Urgences, Nîmes University Hospital, 4 Rue du Professeur Robert Debré, Nîmes, 30029 France; Emergency Department, Hôpital Tenon, Assistance Publique – Hôpitaux de Paris, 4 Rue de la Chine, Paris, 75020 France

**Keywords:** Emergency service, Blood gas monitoring, Transcutaneous, Carbon dioxide, Partial pressure

## Abstract

**Introduction:**

Transcutaneous CO _2_ (PtCO _2_) is a continuous and non-invasive measure recommended by scientific societies in the management of respiratory distress. The objective of this study is to evaluate the correlation between PtCO _2_ and blood pressure of CO _2_ (PaCO _2_) by blood gas analysis in emergency patients with dyspnoea and to determine the factors that interfere in this correlation.

**Methods:**

From January to June 2014, all patients admitted to resuscitation room of the emergency department targeted for arterial blood gases were included prospectively. A sensor measuring the PtCO _2_ was attached to the ear lobe of the patient before the gas analysis. Anamnesis, clinical and laboratory parameters were identified.

**Results:**

90 patients with dyspnoea were included (with 104 pairs of measurements), the median age was 79 years [69-85]. The correlation between PtCO _2_ and PaCO _2_ was R ^2^= 0.83 (p <0.001) but became lower for values of PaCO _2_>60 mm Hg. The mean bias (±SD) between the two methods of measurement (Bland-Altman analysis) was -1.4 mm Hg (±7.7) with limits of agreement of -16.4 to 13.7 mm Hg. In univariate analysis, PaO _2_ interfered in this correlation. After multivariate analysis, the temperature (OR = 3.01, 95% CI = 1.16-7.09) and the PaO _2_ (OR = 1.22, 95% CI = 1.02-1.47) were found to be significant.

**Conclusions:**

In patients admitted in emergency unit for acute respiratory failure, there is a significant correlation between PaCO _2_ and PtCO _2_, mainly for values below 60 mm Hg. The two limiting factors of use are hyperthermia and users training.

## Introduction

In patients with respiratory failure admitted to resuscitation room (RR), the monitoring of arterial blood gases is crucial for diagnosis and therapeutic guidance [1]. The gold standard remains the analysis of arterial blood gases. This implies an arterial puncture that it is invasive, time consuming and giving only punctual results [2,3]. In addition, pending the results of blood gas analysis does not allow for real-time adaptation of oxygen therapy and/or mechanical ventilation. Oxygen saturation by pulse oxymetry (SpO _2_) is widely used as a surrogate of arterial oxygen saturation (SaO _2_) [4]. In mechanically ventilated patients, end tidal CO _2_ (EtCO _2_) allows an indirect reliable continuous assessment of arterial PCO _2_. In non-ventilated patients, assessment of EtCO _2_ is more complex, less accurate and often impossible. The transcutaneous monitoring of the carbon dioxide (PtCO _2_) could represent a good alternative for immediate and continuous assessment of PCO _2_ in RR, especially in non-ventilated patients. The transcutaneous continuous monitoring of the carbon dioxide has been recently recommended [5,6]. In the specific setting of RR, PtCO _2_ has been poorly studied. Numerous studies in infants [7,8] and adults [9-11] have found a good correlation between PaCO _2_ and PtCO _2_. The main objective of this study was to investigate the relationship between measures of PtCO _2_ and PaCO _2_ in resuscitation room patients admitted. The secondary objective was to determine the factors that may disrupt the link between these two parameters.

## Patients and methods

### Setting and study population

This single-center prospective observational study was conducted from January to June 2014 at the emergency department (ED) of Nîmes University Hospital. According to the French Law (Law 88-1138 relative to Biomedical Research of 20 December 1988 modified on 9 August 2004), this non-interventional study did not require approval by an ethics committee nor informed signed consent from patients. It was reviewed and approved by our Institutional Review Board (number: 13/06-02). Moreover, the present study was declared to and approved by the national commission for data processing and civil liberties. All patients provided written informed consent. All adult patients oriented in the RR and with an arterial blood gas during laboratory tests were likely to be included in the study. Exclusion criteria were the realization of venous blood gases and non-compliance of the study protocol (incorrect installation of the sensor or signal abnormality of the monitor or backup).

The PtCO _2_ measurement was performed by a Stow-Severinghaus sensor (tc Sensor 92 by Radiometer™, Copenhagen, Denmark). The sensor was placed on the earlobe heating skin to a temperature of 44 ^∘^C resulting in a dilatation of capillary bed that allows a 20 times faster diffusion of gases (CO _2_ and O _2_) from the skin to the sensor [12]. On the sensor, carbon dioxide reacts with water to form carbonic acid which dissociates into H+ and HCO3 thereby changing pH values. These pH changes are translated into PtCO _2_ value through the Henderson-Hasselbalch formula [13].

Before the study, medical and paramedical staff were trained in the operation and maintenance of the monitor PtCO _2_ type TCM TOSCA monitor (Radiometer™, Copenhagen, Denmark). Patients in whom RR admission was required were included. The PtCO _2_ sensor was attached to the ear lobe of the patient allowing continuous measurement of PtCO _2_. Arterial blood gases and the usual additional assessments were performed at the same time. The medical team was blinded for the value of PtCO _2_.

Medical patient data were collected and computerized after initial collection on paper case report form (CRF). Blood pressure, heart rate and respiratory rate, blood oxygen saturation, Glasgow coma scale, temperature, time for completion of the arterial blood gases, catecholamines use, NIV or tracheal intubation were recorded by the attending physician. Epidemiological characteristics of patients such as admission modalities, length of hospital stay and biological data were collected on the CRF. PtCO _2_ values were automatically saved every ten seconds by the monitor PtCO _2_. The primary outcome variable was concordance between the simultaneous PaCO _2_ and PtCO _2_ values. Sample size calculation was based on the anticipated variation in the differences between the measurements and the required precision. Using previous study [14] for an estimate of the variation between the differences, a sample size of 50 patients gives a precision of ± 0.19 kPa in the limits of agreement. The secondary outcome was to determine the factors that interfere in this correlation.

### Statistical analysis

Patient characteristics are described using qualitative variables (using frequencies and percentages) and quantitative variables (using means and standard deviation or median with interquartile depending on type of distribution). The concordance between PtCO _2_ and PaCO _2_ was evaluated by linear regression (correlation coefficients) and Bland-Altman analysis, which determined bias, precision, and agreement of PtCO _2_ and PaCO _2_, taking the automated analysis in the laboratory as the reference. The Pearson correlation coefficient was used to demonstrate the presence or absence of a relationship between PtCO _2_ and PaCO _2_. Relationships between measurement differences (|*PaCO*_2_-PtCO _2_|) and patient characteristics were investigated by regression analysis. Variables related to the difference between PtCO _2_ and PaCO _2_ in the univariable analysis (defined by p <0.10, forward selection) were further analysed in a multivariable model (analysis of covariance). We introduced in this model PaCO _2_ but did not introduce pH and PtCO _2_ to avoid a collinear bias. Overall model fit was assessed using the Hosmer-Lemeshow test. All statistical tests were 2-sided. A P value less than 0.05 was considered significant for all analyses.

Analyses were performed with the use of R 3.0.2 (R Core Team 2013, R: A language and environment for statistical computing. R Foundation for Statistical Computing, Vienna, Austria). The authors had full access to and take full responsibility for the integrity of the data.

## Results

**Figure 1:**
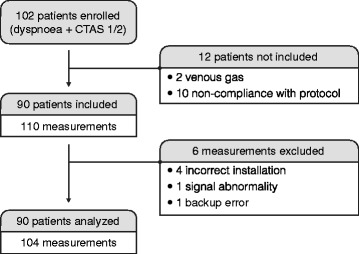
Flow diagram.

**Figure 2:**
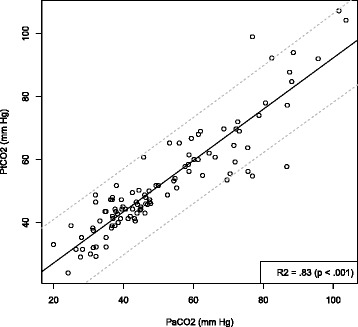
Linear regression between transcutaneous PtcCO _2_ and PaCO _2_. Regression line is the continuous line, the dotted lines show the 95% confidence interval.

**Figure 3:**
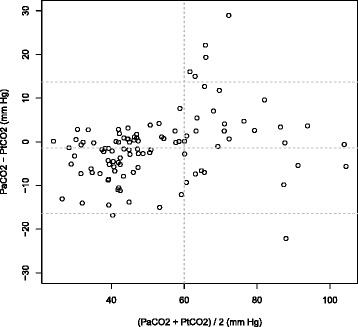
Bland-Altman representation of comparison analysis between PaCO _2_ and PtcCO _2_ vs means of paired measurements.

Between January 2014 and June 2014, 102 patients were screened. Only 90 patients were analyzed with 104 PtCO _2_ values (Figure 1). Table 1 shows the characteristics of the 104 measurements. After linear regression analysis of 104 couples, there was a significant correlation between PaCO _2_ and PtCO _2_ with R ^2^=0.83(*P*<0.001) (Figure 2). The linear regression equation between the two variables was PaCO _2_= 0.81 × + 10.86 PtCO _2_. The Bland-Altman (12) is shown in Figure 3. The mean bias was -1.4 mm Hg (±7.7) and limits of agreement (bias ±1.96 SD) between the two techniques were -16.4 mm Hg and 13.7 mm Hg. The Pearson’s correlation coefficient was 0.94 (95% CI = 0.87-0.94, P <0.001).

**Table 1:** **Patient characteristics**

Male sex, no. (%)	51 (57)
Age, mean (± SD) - year	76 (15)
Past medical history, no. (%)	
Acute pulmonary edema	27 (29)
Chronic obstructive pulmonary disease	27 (29)
Ischemic heart disease	21 (23)
Home oxygen	16 (17)
Clinical data at admission, median (IQR)	
Heart rate - beats/min.	94 (80-110)
Systolic blood pressure - mm Hg	122 (106-144)
Diastolic blood pressure - mm Hg	69 (60-78)
Respiratory rate - breaths/min.	24 (19-28)
Glasgow coma scale	15 (14-15)
Temperature - ^∘^C	37.0 (36.2-37.6)
Laboratory values, median (IQR)	
PaCO _2_ - mm Hg	46.2 (37.6-66.8)
PtCO _2_ - mm Hg	47.2 (42.1-60.0)
PaO _2_ - mm Hg	73.5 (63.0-89.0)
pH	7.37 (7.30-7.43)
HCO _3_ - mEq/L	26.0 (22.8-29.7)
Base excess - mmol/L	1.9 (-1.9-5.8)
Lactate - mmol/L	1.3 (0.7-2.2)
Hemoglobin - g/dL	12.3 (10.9-13.8)
White blood cells - G/L	12.4 (7.9-15.5)
C-reactive protein	41 (8-122)
Glycemia - g/L	1.4 (1.2-1.7)
Brain natriuretic peptide - ng/L	1704 (579-6200)
Diagnosis, no. (%)	
Heart failure	25 (27)
COPD	14 (15)
Pneumonia	42 (46)
Pulmonary embolism	5 (5)
Outcome, no. (%)	
Noninvasive ventilation required	41 (45)
Intubation required	4 (4)
Admitted to hospital	61 (66)
Admitted to ICU	19 (21)
Discharged from ED	10 (11)
Death at the ED	2 (2)
Inpatient mortality	9 (10)

COPD: Chronic obstructive pulmonary disease; ED: Emergency department; ICU: Intensive care unit; IQR: Interquartile range; PaCO _**2**_: Partial pressure of carbon dioxide in the blood; PtCO _**2**_: Transcutaneous partial pressure of carbon dioxide in the blood; PaO _**2**_: Partial pressure of dioxygen in the blood.

For group with PaC02 < 60 mmH, R ^2^=0.70 (P <0.001) and the mean bias was -3.5 mm Hg (±5.0). For group with PaC02 > 60 mmH, R ^2^ = 0.57 (P <0.001) and the mean bias was 4.1 mm Hg (±10.2).

**Figure 4:**
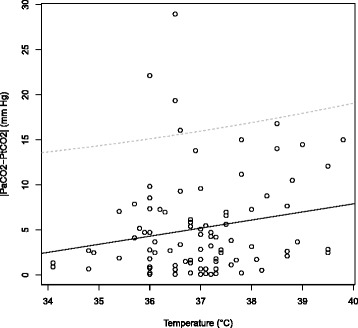
Linear regression between temperature and difference between PaCO _2_ and PtCO _2_ (|*PaCO*_2_-PtCO _2_|). Regression line is the continuous line, the dotted lines show the 95% confidence interval.

In univariate analysis, the only factor associated with a difference between PaCO _2_ and PtCO _2_ was PaO _2_ (Table 2). In multivariate analysis with three explanatory variables (PaCO _2_, PaO _2_, temperature), we found the temperature and the PaO _2_ to be significantly associated with a high difference between PaCO _2_ and PtCO _2_ (Table 2). In this model, 11 observations were deleted because of missing data. This model has a nonsignificant Hosmer-Lemeshow chi-square goodness-of-fit statistic. The higher the temperature of the patient, the higher the difference between PaCO _2_ and PtCO _2_ was significant (Figure 4).

**Table 2:** **Relationships between measurement differences (|PaCO**_**2**_ **-PtCO**_**2**_ **|) and patient characteristics, univariate and multivariate analysis (ANCOVA)**

	**Univariate analysis**		**Multivariate analysis**	
**Variable**	**OR [95% CI]**	**P-value**	**OR [95% CI]**	**P-value**
Sex	1.61 [0.18-14.25]	0.66		
Past medical history				
Acute pulmonary edema	0.29 [0.03-2.96]	0.29		
COPD	0.60 [0.06-6.18]	0.67		
Ischemic heart disease	0.75 [0.06-8.91]	0.82		
Home oxygen	1.01 [0.06-16.77]	0.99		
Heart rate	0.98 [0.94-1.03]	0.40		
Systolic blood pressure	1.01 [0.97-1.05]	0.60		
Diastolic blood pressure	0.97 [0.91-1.03]	0.33		
Respiratory rate	1.01 [0.88-1.18]	0.85		
Temperature	2.45 [0.93-6.49]	0.07	3.01 [1.16-7.80]	0.03
PaCO _2_	1.05 [1.00-1.12]	0.06	1.06 [1.00-1.12]	0.05
PtCO _2_	1.06 [1.00-1.13]	0.06		
PaO _2_	1.21 [1.01-1.45]	0.04	1.22 [1.02-1.47]	0.03
HCO _3_	0.96 [0.80-1.15]	0.64		
Base excess	0.96 [0.82-1.12]	0.60		
Lactate	1.44 [0.43-4.79]	0.54		
Hemoglobin	1.20 [0.74-1.95]	0.45		
White blood cells	0.91 [0.74-1.12]	0.38		
C-reactive protein	1.00 [0.99-1.01]	0.72		
Glycemia	4.70 [0.62-35.58]	0.13		
Brain natriuretic peptide	1.00 [1.00-1.00]	0.82		

ANCOVA: Analysis of covariance; COPD: Chronic obstructive pulmonary disease; CI: Confidence interval; PaCO _**2**_: Partial pressure of carbon dioxide in the blood; PtCO _**2**_: Transcutaneous partial pressure of carbon dioxide in the blood; PaO _**2**_: Partial pressure of dioxygen in the blood.

## Discussion

To our knowledge, this study is the largest cohort of PtCO _2_ measurements in ED. The mean bias was -1.4 mm Hg (±7.7) and the limits of agreement (bias ±1.96 SD) between the two techniques were -16.4 mm Hg and 13.7 mm Hg. There was a significant correlation between PaCO _2_ and PtCO _2_ (R ^2^=0.83, p <0.001). This value is comparable to what was shown in intensive care (R ^2^=0.86, p <0.01) [9] while other studies have found better correlation (R ^2^ coefficient between 0.91 and 0.99 [15-17]). First, this difference can be explained by the recruitment of our patients, performed only in RR with acute respiratory failure. Because a majority of our patients were non-intubated, our results highlight the feasibility and the potential interest of PtCO2 as EtCO2 cannot easily be monitored in non-intubated patients. Indeed, the extreme values of PaCO _2_ and high PaCO _2_ values were reported as possibly interfering in the correlation-PtCO _2_ PaCO _2_ [9,18-20]. Second, this difference may result from the use of the device by a large number of doctors. Calibration, sensor placement and latency to reach the plateau value of PtCO _2_ may differ from one doctor to another. This may lead to poorer reproducibility; some operators use the apparatus less frequently. However, this reflects our center daily practice and occurs when there is any change of material. Third, our population was more likely to have breathing disorders and therefore agitation and/or sweating leading to mobilization of the sensor which may disrupt the measurement. Our study found a value PtCO _2_ generally greater than the value of PaCO _2_ measured. Indeed, linear regression equation is PtCO _2_ = 0.81 × + 10.86 PaCO _2_. This overestimation is in accordance with available literature [9,21,22]. This overestimation may have implications for patients requiring non-invasive ventilation and with no blood gas reference. Thus, the recommendations highlight the need to conduct a gas analysis to verify the correlation between the PaCO _2_ and PtCO _2_ [5]. This parameter is particularly important to consider given that the analysis of Bland-Altman reveals a poorer correlation values PtCO _2_>60 mm Hg. The value of the mean bias reported in our study corresponds to those found in the literature (-1.4 to 4.6 mm Hg) [15,23,24]. This dispersion of the correlation for high values of PaCO _2_ was previously reported. The accuracy of PtCO _2_ seems to be restricted to patients with PaCO _2_ values of <56 mm Hg [25]. One explanation for this poor correlation with the clinical manifestations of hypercapnia (excessive sweating and vasodilatation) leading to a lower diffusion of carbon dioxide [25]. In our study, after multivariate analysis, only the temperature was associated with a poor correlation between PaCO _2_ and PtCO _2_ (HR = 1.15 [0.2-2.10], p = 0.018). The notion that the temperature can influence the correlation had been raised by Rodriguez et al. [26]. The linear regression analysis reveals that the higher is the body temperature, the greater the difference between the PaCO _2_ and PtCO _2_ is important. This poor correlation can be explained by the fact that the higher the patient’s temperature, the greater the difference between the temperature sensor (44 ^∘^C) is low, resulting in small changes in local perfusion and thus altering the local production of CO _2_. This hypothesis follows directly from the operating principle of the sensor [5]. It could also be hypothesized that a high body temperature promotes sweating and vasodilatation making far more difficult sensor measurement. Finally, a low blood pressure can also be a cause a poor correlation [27]. This hypothesis cannot be confirmed by the present data, has few patients has shock criteria. Similarly, the assumption that the pH may explain poor correlation [20] is not confirmed in our study in the multivariate analysis.

### Limitations

First, our study has few patients with hemodynamic instability requiring the establishment of intravenous fluids or vasopressor support. It is therefore difficult to conclude to any contribution from the low-speed on the correlation between PaCO _2_ and PtCO _2_. Several studies have shown that the correlation between the PtCO _2_ PaCO _2_ and is not affected by the use of catecholamine but by dermal vasoconstriction secondary to a state of shock [9,22,26]. Secondly, body mass index (BMI) has not been measured over the study. Several studies reported conflicting conclusions regarding the influence of the thickness of the skin, indirectly estimated by BMI, on the diffusion of CO _2_ to the skin and therefore the values of PcCO _2_ [9,22,24,25]. Third, the majority of patients had received only blood gases during the treatment, not for obtaining intra-individual correlations between different values PtCO _2_ and PaCO _2_. This analysis was important to be able to easily predict PaCO _2_ values from continuous measurement of PtCO _2_ in patients requiring several hours of surveillance [26,28].

## Conclusions

In patients admitted in emergency unit for acute respiratory failure, there is a significant correlation between PaCO _2_ and PtCO _2_, mainly for values below 60 mm Hg. The two limiting factors of use are hyperthermia and users training.

## Key messages

- In patients admitted in emergency unit for acute respiratory failure, there is a significant correlation between PaCO _2_ and PtCO _2_.
- This correlation is comparable to what was shown in intensive care.
- The two limiting factors of use are hyperthermia and users training.

## Abbreviations

ANCOVA: Analysis of covariance
BMI: Body mass index
CI: Confidence interval
COPD: Chronic obstructive pulmonary disease
CRF: Case report form
ED: Emergency department
EtCO _2_: End tidal CO _2_
ICU: Intensive care unit
IQR: Interquartile range
OR: Odds ratio
RR: Resuscitation room
PaCO _2_: Partial pressure of carbon dioxide in the blood
PtCO _2_: Transcutaneous partial pressure of carbon dioxide in the blood
PaO _2_: Partial pressure of dioxygen in the blood
SaO _2_: Arterial oxygen saturation
SpO _2_: Oxygen saturation by pulse oxymetry

## References

[1] Rady MY (2005). Bench-to-bedside review: Resuscitation in the emergency department. Crit Care.

[2] Burri E, Potocki M, Drexler B, Schuetz P, Mebazaa A, Ahlfeld U (2011). Value of arterial blood gas analysis in patients with acute dyspnea: an observational study. Crit Care.

[3] Bobbia X, Grandpierre RG, Claret PG, Moreau A, Pommet S, Bonnec JM (2013). Ultrasound guidance for radial arterial puncture: a randomized controlled trial. Am J Emerg Med.

[4] Silversides JA, Ferguson ND (2013). Clinical review: Acute respiratory distress syndrome - clinical ventilator management and adjunct therapy. Crit Care.

[5] Restrepo RD, Hirst KR, Wittnebel L, Wettstein R (2012). AARC clinical practice guideline: transcutaneous monitoring of carbon dioxide and oxygen: 2012. Respir Care.

[6] Brochard L, Martin GS, Blanch L, Pelosi P, Belda FJ, Jubran A (2012). Clinical review: Respiratory monitoring in the ICU - a consensus of 16. Crit Care.

[7] Monaco F, Nickerson BG, McQuitty JC (1982). Continuous transcutaneous oxygen and carbon dioxide monitoring in the pediatric ICU. Crit Care Med.

[8] O’Connor TA, Grueber R (1998). Transcutaneous measurement of carbon dioxide tension during long-distance transport of neonates receiving mechanical ventilation. J Perinatol.

[9] Bendjelid K, Schütz N, Stotz M, Gerard I, Suter PM, Romand JA (2005). Transcutaneous PCO2 monitoring in critically ill adults: clinical evaluation of a new sensor. Crit Care Med.

[10] Delerme S, Montout V, Goulet H, Arhan A, Devilliers C, Le Saché F (2012). Concordance between transcutaneous and arterial measurements of carbon dioxide in an ED. Am J Emerg Med.

[11] Gancel PE, Roupie E, Guittet L, Laplume S, Terzi N (2011). Accuracy of a transcutaneous carbon dioxide pressure monitoring device in emergency room patients with acute respiratory failure. Intensive Care Med.

[12] Severinghaus JW, Astrup P, Murray JF (1998). Blood gas analysis and critical care medicine. Am J Respir Crit Care Med.

[13] Kellum JA (2000). Determinants of blood pH in health and disease. Crit Care.

[14] Heuss LT, Chhajed PN, Schnieper P, Hirt T, Beglinger C (2004). Combined pulse oximetry/cutaneous carbon dioxide tension monitoring during colonoscopies: pilot study with a smart ear clip. Digestion.

[15] Storre JH, Steurer B, Kabitz HJ, Dreher M, Windisch W (2007). Transcutaneous PCO2 monitoring during initiation of noninvasive ventilation. Chest.

[16] McVicar J, Eager R (2009). Validation study of a transcutaneous carbon dioxide monitor in patients in the emergency department. Emerg Med J.

[17] Cox M, Kemp R, Anwar S, Athey V, Aung T, Moloney ED (2006). Non-invasive monitoring of CO2 levels in patients using NIV for AECOPD. Thorax.

[18] McLellan PA, Goldstein RS, Ramcharan V, Rebuck AS (1981). Transcutaneous carbon dioxide monitoring. Am Rev Respir Dis.

[19] Tobias JD (2009). Transcutaneous carbon dioxide monitoring in infants and children. Paediatr Anaesth.

[20] Versmold HT, Linderkamp O, Holzmann M, Strohhacker I, Riegel K (1979). Transcutaneous monitoring of PO2 in newborn infants: where are the limits? Influence of blood pressure, blood volume, blood flow, viscosity, and acid base state. Birth Defects Orig Artic Ser.

[21] Rosner V, Hannhart B, Chabot F, Polu JM (1999). Validity of transcutaneous oxygen/carbon dioxide pressure measurement in the monitoring of mechanical ventilation in stable chronic respiratory failure. Eur Respir J.

[22] Janssens JP, Howarth-Frey C, Chevrolet JC, Abajo B, Rochat T (1998). Transcutaneous PCO2 to monitor noninvasive mechanical ventilation in adults: assessment of a new transcutaneous PCO2 device. Chest.

[23] Lang CJ (1998). Apnea testing guided by continuous transcutaneous monitoring of partial pressure of carbon dioxide. Crit Care Med.

[24] Maniscalco M, Zedda A, Faraone S, Sofia M, Carratù P (2008). Evaluation of a transcutaneous carbon dioxide monitor in severe obesity. Intensive Care Med.

[25] Cuvelier A, Grigoriu B, Molano LC, Muir JF (2005). Limitations of transcutaneous carbon dioxide measurements for assessing long-term mechanical ventilation. Chest.

[26] Rodriguez P, Lellouche F, Aboab J, Buisson CB, Brochard L (2006). Transcutaneous arterial carbon dioxide pressure monitoring in critically ill adult patients. Intensive Care Med.

[27] Belenkiy S, Ivey KM, Batchinsky AI, Langer T, Necsoiu C, Baker W (2013). Noninvasive carbon dioxide monitoring in a porcine model of acute lung injury due to smoke inhalation and burns. Shock.

[28] Randerath WJ, Stieglitz S, Galetke W, Anduleit N, Treml M, Schäfer T (2010). Evaluation of a system for transcutaneous long-term capnometry. Respiration.

