# Erratum: Concordance and limits between transcutaneous and arterial carbon dioxide pressure in emergency department patients with acute respiratory failure: a single-center, prospective, and observational study

**DOI:** 10.1186/s13049-015-0154-7

**Published:** 2015-10-06

**Authors:** Xavier Bobbia, Pierre-Géraud Claret, Ludovic Palmier, Michaël Robert, Romain Genre Granpierre, Claire Roger, Justin Yan, Patrick Ray, Mustapha Sebbane, Laurent Muller, Jean-Emmanuel de La Coussaye

**Affiliations:** Pôle Anesthésie Réanimation Douleur Urgences, Nîmes University Hospital, 4 Rue du Professeur Robert Debré 30029 Nîmes, Nîmes, France; Division of Emergency Medicine, Department of Medicine, London Health Sciences Centre and The Schulich School of Medicine and Dentistry, The University of Western Ontario, London, Ontario Canada; Emergency Department, Hôpital Tenon, Assistance Publique - Hôpitaux de Paris, 4 Rue de la Chine 75020 Paris, Paris, France

**Keywords:** Emergency service, Blood gas monitoring, Transcutaneous, Carbon dioxide, Partial pressure

## Abstract

**Abstract:**

After publication of this article (Scand J Trauma Resusc Emerg Med 23:40, 2015), it came to light that an earlier version had been published in error. This erratum contains the correct version of the article, which incorporates revisions made in response to reviewer comments. Additionally, one of the authors was inadvertently omitted from the author list. This author, Justin Yan, has been included in the corrected author list above.

**Background:**

Transcutaneous CO_2_ (PtCO_2_) is a continuous and non-invasive measure recommended by scientific societies in the management of respiratory distress. The objective of this study was to evaluate the correlation between PtCO_2_ and arterial partial pressure of CO_2_ (PaCO_2_) by arterial blood gas analysis in emergency patients with dyspnoea, and to determine the factors that interfere with this correlation.

**Methods:**

From January to June 2014, all adult patients admitted to the RR with dyspnoea during business hours were included in the study if arterial blood gas measurements were indicated. A sensor measuring the PtCO_2_ was attached to the ear lobe of the patient before the gas analysis. Anamnesis, clinical and laboratory parameters were identified.

**Results:**

Ninety patients with dyspnoea were included (104 pairs of measurements). The median (IQR) age was 79 years (69 – 85). The correlation between PtCO_2_ and PaCO_2_ was R^2^ =.83 (*p*<.001) but became lower for values of PaCO_2_ above 60 mm Hg. The mean bias (± SD) between the two methods of measurement (Bland-Altman analysis) was −1.4 mm Hg (± 7.7) with limits of agreement from −16.4 to 13.7 mm Hg. In univariate analysis, PaO_2_ interfered with this correlation. After multivariate analysis, temperature (OR = 3.01; 95 % CIs [1.16, 7.80]) and PaO_2_ (OR = 1.22; 95 % CIs [1.02, 1.47]) significantly interfered with this correlation.

**Conclusions:**

There is a significant correlation between PaCO_2_ and PtCO_2_ values for patients admitted to the emergency department for acute respiratory failure. One limiting factor to routine use of PtCO_2_ measurements in the emergency department is the presence of hyperthermia.

## Background

This is a corrected version of the previously published article [1]. Arterial blood gas monitoring is crucial for management of patients with respiratory failure [2]. The gold standard technique involves an arterial puncture which is invasive, time-consuming, and only gives results at one point in time [3, 4]. Moreover, the delay in waiting for the results of blood gas analysis does not allow for real-time adaptation of oxygen therapy or mechanical ventilation. Oxygen saturation by pulse oximetry (SpO_2_) is widely used as a surrogate of arterial oxygen saturation (SaO_2_) [5]. Similarly, end tidal CO_2_ (EtCO_2_) allows for an indirect, but reliable and continuous assessment of arterial pCO_2_ for mechanically ventilated patients. However, for non-ventilated patients, assessment of EtCO_2_ is more complex, less accurate, and often impossible. For these patients, the recently recommended [6, 7] transcutaneous monitoring of carbon dioxide (PtCO_2_) could represent an alternative for immediate and continuous assessment of pCO_2_. Numerous studies of both children [8, 9] and adults [10–12] have found a good correlation between PaCO_2_ and PtCO_2_. Yet in the specific setting of the emergency department (ED) resuscitation room (RR), PtCO_2_ has been poorly studied. The main objective of this study was to investigate the relationship between measures of PtCO_2_ and PaCO_2_ for patients admitted to the ED RR. The secondary objective was to determine the variables that may disrupt the link between PtCO_2_ and PaCO_2_.

## Methods

### Setting

We conducted this single-center prospective observational study from January to June 2014 in the ED of Nîmes University Hospital, France. This study was reviewed and approved by our Institutional Review Board (number: 13/06–02) and was declared to and approved by the national commission for data processing and civil liberties. All patients provided written informed consent. This study is in compliance with the Helsinki Declaration.

### Study population

All adult patients admitted to the RR with dyspnoea during business hours (from 9:00 to 17:00, weekend excluded) were included in the study if arterial blood gas measurements were indicated. In our ED, patients are admitted to the RR if they are level 1 or level 2 according to the Canadian Triage and Acuity Scale (CTAS). Thus, patients with dyspnoea are admitted to the RR if they suffered from severe respiratory distress, asthma, or important dyspnoea. Definition of CTAS level 1 and level 2 for dyspnoea are specified in Appendix 1. Exclusion criteria were incorrect installation of the sensor, signal abnormality on the monitor, and backup error on the memory of the device.

### Measurement

The PtCO_2_ measurement was performed by a Stow-Severinghaus sensor (tc Sensor 92 by Radiometer™, Copenhagen, Denmark). The sensor heats skin to a temperature of 44 °C resulting in a dilatation of the capillary bed that allows for diffusion of gases (CO_2_ and O_2_) [13]. On the sensor, carbon dioxide reacts with water to form carbonic acid which dissociates into H ^+^ and HCO$_{3}^{-}$, thereby changing pH values. These pH changes are translated into PtCO_2_ value through the Henderson-Hasselbalch formula [14]. Medical and paramedical staff were trained in the operation and maintenance of the PtCO_2_ TOSCA monitor (Radiometer™, Copenhagen, Denmark) before the study commenced. For included patients, the PtCO_2_ sensor was attached to the ear lobe of the patient allowing for continuous measurement of PtCO_2_. After stabilization of the monitor to obtain a good signal, arterial blood gases and PtCO_2_ measurements were performed simultaneously. The medical team was blinded to the value of PtCO_2_ measured.

### Outcomes

The primary outcome was concordance between the simultaneous PaCO_2_ and PtCO_2_ values. The sample size calculation was based on the anticipated variation in the differences between the measurements and the required precision. Using a previous study [15] for an estimate of the variation between the differences, a sample size of 50 patients gave a precision of ± 0.19 kPa as the limits of agreement. The secondary outcome was to determine the factors that interfere with this correlation. PtCO_2_ values were automatically saved every ten seconds by the monitor. Medical patient data were collected and entered into an electronic database after initial collection on paper case report forms (CRF). Blood pressure, heart rate, respiratory rate, blood oxygen saturation, Glasgow coma scale, temperature, time to completion of arterial blood gases, catecholamine use, and non-invasive ventilation or tracheal intubation were recorded by the attending physician. Characteristics of patients such as ED arrival modalities, hospital length of stay, and biological data were collected on the CRF.

### Statistical analysis

Patient characteristics were described using qualitative (frequencies and percentages) or quantitative variables (means and standard deviations or median with interquartile ranges - depending on type of distribution) where appropriate. The concordance between PtCO_2_ and PaCO_2_ was evaluated by linear regression (correlation coefficients) and Bland-Altman analysis, which determined bias, precision, and agreement of PtCO_2_ and PaCO_2_, taking the automated analysis in the laboratory as the reference standard. The Pearson correlation coefficient was used to demonstrate the presence or absence of a relationship between PtCO_2_ and PaCO_2_. Relationships between measurement differences (|PaCO_2_- PtCO_2_ |) and patient characteristics were investigated by regression analysis. Variables related to the difference between PtCO_2_ and PaCO_2_ in the univariate analysis (defined by *p*<.1, forward selection) were further analyzed in a multivariate model (analysis of covariance). We included PaCO_2_ in this model but did not included pH or PtCO_2_ to avoid a collinear bias. Overall model fit was assessed using the Hosmer-Lemeshow test. All statistical tests were two sided. A *p*-value less than.05 was considered significant for all analyses.

Analyses were performed with the use of R 3.0.2 (R Core Team 2013, R: A language and environment for statistical computing. R Foundation for Statistical Computing, Vienna, Austria). The authors had full access to and take full responsibility for the integrity of the data.

## Results

Between January 2014 and June 2014, 102 patients were screened for eligibility. Ninety patients were included and analyzed with 104 PtCO_2_ values (Fig. 1). Table 1 shows the patient characteristics corresponding to the 104 measurements. After linear regression analysis of 104 couples of measurements, we found a significant correlation between PaCO_2_ and PtCO_2_ with R^2^ =.83 (*p*<.001) (Fig. 2). The linear regression equation between the two variables was PaCO_2_ = (0.81 × PtCO_2_) +10.86. The Bland-Altman analysis is shown in Fig. 3. The mean bias was −1.4 mm Hg (± 7.7) and the limits of agreement (bias ± 1.96 SD) between the two techniques were −16.4 mm Hg and 13.7 mm Hg. The Pearson’s correlation coefficient was.94 (95 % CIs [0.87, 0.94]; *p*<.001). For the group with PaC02 < 60 mm Hg, R^2^ =.70 (*p*<.001) and the mean bias was −3.5 mm Hg (± 5.0). For the group with PaC02 > 60 mm Hg, R^2^ =.57 (*p*<.001) and the mean bias was 4.1 mm Hg (± 10.2).

**Fig. 1:**
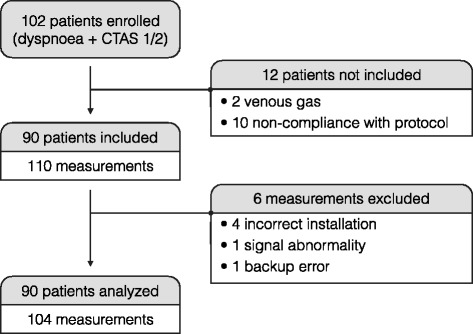
Flow diagram

**Fig. 2:**
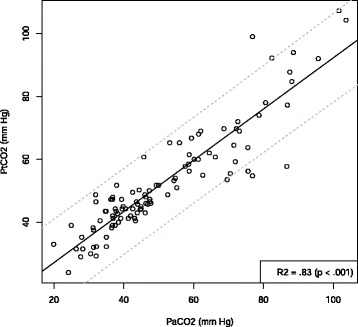
Linear regression between transcutaneous PtcCO_2_ and PaCO_2_. Regression line is the continuous line, the dotted lines show the 95 % confidence interval

**Fig. 3:**
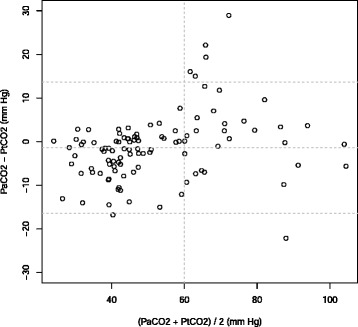
Bland-Altman representation of comparison analysis between PaCO_2_ and PtcCO_2_ vs means of paired measurements

**Table 1:** Patients’ characteristics

Male sex, no. (%)	51 (57)
Age, mean (± SD) - year	76 (15)
Past medical history, no. (%)	
Acute pulmonary edema	27 (29)
Chronic obstructive pulmonary disease	27 (29)
Ischemic heart disease	21 (23)
Home oxygen	16 (17)
Clinical data at admission, median (IQR)	
Heart rate - beats/min.	94 (80–110)
Systolic blood pressure - mm Hg	122 (106–144)
Diastolic blood pressure - mm Hg	69 (60–78)
Respiratory rate - breaths/min.	24 (19–28)
Glasgow coma scale	15 (14–15)
Temperature - °C	37.0 (36.2–37.6)
Laboratory values, median (IQR)	
PaCO_2_ - mm Hg	46.2 (37.6–66.8)
PtCO_2_ - mm Hg	47.2 (42.1–60.0)
PaO_2_ - mm Hg	73.5 (63.0–89.0)
pH	7.37 (7.30–7.43)
HCO_3_ - mEq/L	26.0 (22.8–29.7)
Base excess - mmol/L	1.9 (-1.9–5.8)
Lactate - mmol/L	1.3 (0.7–2.2)
Hemoglobin - g/dL	12.3 (10.9–13.8)
White blood cells - G/L	12.4 (7.9–15.5)
C-reactive protein	41 (8–122)
Glycemia - g/L	1.4 (1.2–1.7)
Brain natriuretic peptide - ng/L	1704 (579–6200)
Diagnosis, no. (%)	
Heart failure	25 (27)
COPD	14 (15)
Pneumonia	42 (46)
Pulmonary embolism	5 (5)
Outcome, no. (%)	
Noninvasive ventilation required	41 (45)
Intubation required	4 (4)
Admitted to hospital	61 (66)
Admitted to ICU	19 (21)
Discharged from ED	10 (11)
Death at the ED	2 (2)
Inpatient mortality	9 (10)

In the univariate analysis, the only factor associated with a difference between PaCO_2_ and PtCO_2_ was PaO_2_ (Table 2). In multivariate analysis with three explanatory variables (PaCO_2_, PaO_2_, temperature), we found the temperature and the PaO_2_ to be significantly associated with a large difference between PaCO_2_ and PtCO_2_ (Table 2). The higher the temperature of the patient, the greater the difference between PaCO_2_ and PtCO_2_ (Fig. 4). We developed this model on a data set of 93 measurements (11 observations were exluded due to missingness). This model had a non-significant Hosmer-Lemeshow chi-square goodness-of-fit statistic.

**Fig. 4:**
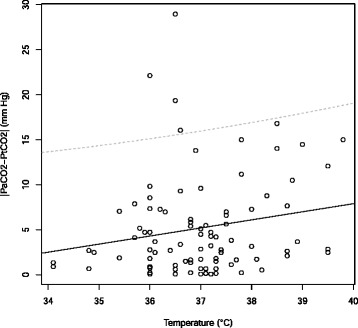
Linear regression between temperature and difference between PaCO_2_ and PtCO_2_ (|PaCO_2_-PtCO _2_|). Regression line is the continuous line, the dotted lines show the 95 % confidence interval

**Table 2:** Relationships between measurement differences (|PaCO2-PtCO2 |) and patient characteristics using univariate and multivariate analysis (ANCOVA)

	Univariate analysis		Multivariate analysis	
Variable	OR [95 % CIs]	*P*-value	OR [95 % CIs]	*P*-value
Sex	1.61 [0.18, 14.25]	.66		
Past medical history				
Acute pulmonary	0.29 [0.03, 2.96]	.29		
edema				
COPD	0.60 [0.06, 6.18]	.67		
Ischemic heart	0.75 [0.06, 8.91]	.82		
disease				
Home oxygen	1.01 [0.06, 16.77]	.99		
Heart rate	0.98 [0.94, 1.03]	.40		
Systolic blood	1.01 [0.97, 1.05]	.60		
pressure				
Diastolic blood	0.97 [0.91, 1.03]	.33		
pressure				
Respiratory rate	1.01 [0.88, 1.18]	.85		
Temperature	2.45 [0.93, 6.49]	.07	3.01 [1.16, 7.80]	.03
PaCO_2_	1.05 [1.00, 1.12]	.06	1.06 [1.00, 1.12]	.05
PtCO_2_	1.06 [1.00, 1.13]	.06		
PaO_2_	1.21 [1.01, 1.45]	.04	1.22 [1.02, 1.47]	.03
HCO_3_	0.96 [0.80, 1.15]	.64		
Base excess	0.96 [0.82, 1.12]	.60		
Lactate	1.44 [0.43, 4.79]	.54		
Hemoglobin	1.20 [0.74, 1.95]	.45		
White blood cells	0.91 [0.74, 1.12]	.38		
C-reactive protein	1.00 [0.99, 1.01]	.72		
Glycemia	4.70 [0.62, 35.58]	.13		
Brain natriuretic	1.00 [1.00, 1.00]	.82		
vpeptide				

## Discussion

To our knowledge, our study is the largest cohort of PtCO_2_ measurements conducted in the ED. The mean bias was −1.4 mm Hg (± 7.7) and the limits of agreement (bias ± 1.96 SD) between the two techniques were −16.4 mm Hg and 13.7 mm Hg. There was a significant correlation between PaCO_2_ and PtCO_2_ (R^2^ =.83; *p*<.001). Because most of our patients were non-intubated, our results highlight the feasibility and the potential benefit of measuring PtCO2 since EtCO2 cannot easily be monitored in non-intubated patients. The correlation coefficient in our study was comparable to what was shown in a previous intensive care study (R^2^ =.86; *p*<.01) [10]. However, other studies have found a stronger correlation (R^2^ coefficient, ranged between.91 and.99 [16–18]). We assume this difference is not a consequence of the use of various devices since most of the studies were completed with a Radiometer™device. This difference can be explained by the selection of our patients, as only those in the RR with acute respiratory failure were included. Indeed, the high and extreme PaCO_2_ values were reported as possibly interfering with the correlation between PtCO_2_ and PaCO_2_ [10, 19–21]. In a study by Delerme et al. [11], patients had a lower PaCO2 than in our study (39 mm Hg vs. 46, respectively). Secondly, this difference may result from the use of the device by many physicians. Calibration, sensor placement and latency to reach the plateau value of PtCO_2_ may differ from one physician to another. Because some operators used the monitor less frequently, this may have led to poorer reproducibility. However, it also reflects our center’s daily practice and this issue may occur with any change of device. Thirdly, we did not correct the arterial blood gases according to the patient’s temperature and this may explain a portion of the increased difference. Finally, our population was more likely to have significant dyspnoea and therefore agitation or diaphoresis leading to movement of the sensor may have led to inaccurate measurements. Indeed, in the Gancel et al. study [12] where the difference was lower, the exclusion criteria were very rigorous. Authors did not study patients with status epilepticus, confusion or agitation. According to the authors, these criteria may have led to the exclusion of some patients with severe hypercapnia.

Our study found that PtCO_2_ values were generally greater than PaCO_2_ values. Indeed, our linear regression equation is PaCO_2_ = (0.81 × PtCO_2_) + 10.86. This overestimation is in accordance with available literature [10, 22, 23] and may have implications for patients requiring non-invasive ventilation and with no arterial blood gas reference. Thus, the recommendations highlight the need to conduct an arterial blood gas analysis to support the correlation between PaCO_2_ and PtCO_2_ values [6]. This issue is important given that the Bland-Altman analysis reveals a poorer correlation for PtCO_2_ values above 60 mm Hg. The value of the mean bias reported in our study corresponds to those found in the literature (−1.4 to 4.6 mm Hg) [16, 24, 25]. The decrease of the correlation for high PaCO_2_ values has been previously reported. The accuracy of PtCO_2_ seems to be better for patients with PaCO_2_ values below 56 mm Hg [26]. One explanation for this poor correlation is that the clinical manifestations of hypercapnia (excessive sweating and vasodilatation) leads to a lower diffusion of carbon dioxide [26]. In our study, after multivariate analysis, temperature was associated with a poor correlation between PaCO_2_ and PtCO_2_ (OR = 3.01; 95 % CIs [1.16, 7.80]; *p*=.03). The issue that the temperature can influence the correlation has been raised by Rodriguez et al. [27]. Our linear regression analysis revealed that the higher the body temperature, the greater the difference between PaCO_2_ and PtCO_2_ values. This poor correlation can be explained by the fact that asthe patient’s temperature increased, the difference between the patient and the temperature sensor (44 °C) decreased, resulting in small changes in local perfusion and production of CO_2_. This hypothesis follows directly from the operating principle of the sensor [6]. It could also be hypothesized that a high body temperature promotes sweating and vasodilatation making the sensor’s measurement more inaccurate. Finally, a low blood pressure could also be a cause of a poor correlation between PaCO_2_ and PtCO_2_ [28]. Unfortunately, this hypothesis cannot be confirmed by our data because few patients had shock criteria. Similarly, the assumption that the pH may explain a poor correlation [21] cannot not be confirmed in our study with the multivariate analysis.

### Limitations

Although several studies have found a poor correlation between PaCO_2_ and PtCO_2_ in patients with shock who are treated with catecholamines [10], we did not analyze this particular relationship. Indeed, we included few patients with hemodynamic instability requiring the administration of intravenous fluids or vasopressor support. It is therefore difficult to assess the impact of decreased circulation on the correlation between PaCO_2_ and PtCO_2_. Several studies have shown that the correlation is not affected by catecholamines but by dermal vasoconstriction secondary to a state of shock [23, 27].

Secondly, body mass index (BMI) was not measured in our study. Several studies reported conflicting conclusions regarding the influence of skin thickness, indirectly estimated by BMI, on the CO_2_ diffusion to the skin and therefore on the PaCO_2_ values [10, 23, 25, 26]. However, there is no correlation between BMI and the skin on the earlobe, where the sensor was fixed [29].

Finally, one subject that remains to be explored is the intra-individual correlation. Most of the patients had only one arterial blood gas measurement during their management in the RR, which was inadequate for obtaining intra-individual correlations between different PtCO_2_ and PaCO_2_ values. This analysis would be important to predict PaCO_2_ values from continuous measurement of PtCO_2_, especially for patients requiring several hours of monitoring [27, 30].

## Conclusions

There is a significant correlation between PaCO_2_ and PtCO_2_ values for patients admitted to the ED for acute respiratory failure. This correlation is particularly accurate for values below 60 mm Hg. One limiting factor to routine use of PtCO_2_ measurements in the ED is the presence of hyperthermia.

## Key messages

- There is a significant correlation between PaCO_2_ and PtCO_2_ values for patients admitted to the emergency department for acute respiratory failure.
- This correlation is comparable to that which has been shown in intensive care.
- One limiting factor to the use of PtCO_2_ measurements in the ED is the presence of hyperthermia.

## Appendix 1: Definition of Canadian triage and acuity scale (CTAS) level 1 and level 2 for dyspnoea

### Patients with dyspnoea and CTAS Level 1:

Severe respiratory distress: serious intracranial events, pneumothorax, near death asthma (unable to speak, cyanosis, lethargic/confused, tachycardia/bradycardia, arterial oxygen saturation below 90 %), chronic obstructive pulmonary disease exacerbations, cardiac heart failure, anaphylaxis and severe metabolic disturbances (renal failure, diabetic keto-acidosis).

### Patients with dyspnoea and CTAS Level 2:

Asthma: severe asthma defined with a combination of objectives measures and clinical factors which relate to the severity of symptoms, vital signs and history of previous severe episodes. If the forced expiratory volume in 1 second or peak expiratory flow rate are below 40 % predicted or previous best, the patient is considered severe.

Dyspnea: this is subjective and may correlate poorly with lung function or deficits in oxygen uptake and delivery. Depending on the age, previous history and physical assessment one may not be able to distinguish between asthma chronic obstructive pulmonary disease, cardiac heart failure, pulmonary embolism, pneumothorax, pneumonia, croup, epiglottitis, anaphylaxis or a combination of problems.

## Abbreviations

ANCOVA: Analysis of covariance
BMI: Body mass index
CI: Confidence interval
CTAS: Canadian triage and acuity scale
COPD: Chronic obstructive pulmonary disease
CRF: Case report form
ED: Emergency department
EtCO_2_: End tidal CO_2_
ICU: Intensive care unit
IQR: Interquartile range
OR: Odds ratio
RR: Resuscitation room
PaCO_2_: Partial pressure of carbon dioxide in the blood
PtCO_2_: Transcutaneous partial pressure of carbon dioxide in the blood
PaO_2_: Partial pressure of dioxygen in the blood
SaO_2_: Arterial oxygen saturation
SpO_2_: Oxygen saturation by pulse oxymetry
